# Minimizing acquisition-related radiomics variability by image resampling and batch effect correction to allow for large-scale data analysis

**DOI:** 10.1007/s00330-020-07174-0

**Published:** 2020-09-09

**Authors:** Marta Ligero, Olivia Jordi-Ollero, Kinga Bernatowicz, Alonso Garcia-Ruiz, Eric Delgado-Muñoz, David Leiva, Richard Mast, Cristina Suarez, Roser Sala-Llonch, Nahum Calvo, Manuel Escobar, Arturo Navarro-Martin, Guillermo Villacampa, Rodrigo Dienstmann, Raquel Perez-Lopez

**Affiliations:** 1 Radiomics Group, Vall d’Hebron Institute of Oncology (VHIO), Hospital Universitari Vall d’Hebron, Vall d’Hebron Barcelona Hospital Campus (Spain), Barcelona, Spain; 2 Medical Physics and Radiation Protection Department, Catalan Institute of Oncology (ICO), Duran i Reynals Hospital, Barcelona, Spain; 3 Radiology Department, Bellvitge University Hospital, Barcelona, Spain; 4 Radiology Department, Vall d’Hebron University Hospital, Barcelona, Spain; 5 Medical Oncology, Vall d’Hebron Institute of Oncology (VHIO), Hospital Universitari Vall d´Hebron, Vall d’Hebron Barcelona Hospital Campus (Spain), Barcelona, Spain; 6 Department of Biomedicine, Faculty of Medicine, University of Barcelona, Barcelona, Spain; 7 Radiation Oncology Department, Catalan Institute of Oncology (ICO), Duran i Reynals Hospital, Barcelona, Spain; 8 Oncology Data Science (ODysSey) Group, Vall d’Hebron Institute of Oncology (VHIO), Hospital Universitari Vall d’Hebron, Vall d’Hebron Barcelona Hospital Campus (Spain), Barcelona, Spain

**Keywords:** Radiologic phantom, X-ray computed tomography, Image processing, Metastasis

## Abstract

**Objective:**

To identify CT-acquisition parameters accounting for radiomics variability and to develop a post-acquisition CT-image correction method to reduce variability and improve radiomics classification in both phantom and clinical applications.

**Methods:**

CT-acquisition protocols were prospectively tested in a phantom. The multi-centric retrospective clinical study included CT scans of patients with colorectal/renal cancer liver metastases. Ninety-three radiomics features of first order and texture were extracted. Intraclass correlation coefficients (ICCs) between CT-acquisition protocols were evaluated to define sources of variability. Voxel size, ComBat, and singular value decomposition (SVD) compensation methods were explored for reducing the radiomics variability. The number of robust features was compared before and after correction using two-proportion *z* test. The radiomics classification accuracy (*K*-means purity) was assessed before and after ComBat- and SVD-based correction.

**Results:**

Fifty-three acquisition protocols in 13 tissue densities were analyzed. Ninety-seven liver metastases from 43 patients with CT from two vendors were included. Pixel size, reconstruction slice spacing, convolution kernel, and acquisition slice thickness are relevant sources of radiomics variability with a percentage of robust features lower than 80%. Resampling to isometric voxels increased the number of robust features when images were acquired with different pixel sizes (*p* < 0.05). SVD-based for thickness correction and ComBat correction for thickness and combined thickness–kernel increased the number of reproducible features (*p* < 0.05). ComBat showed the highest improvement of radiomics-based classification in both the phantom and clinical applications (*K*-means purity 65.98 vs 73.20).

**Conclusion:**

CT-image post-acquisition processing and radiomics normalization by means of batch effect correction allow for standardization of large-scale data analysis and improve the classification accuracy.

**Key Points:**

*• The voxel size (accounting for the pixel size and slice spacing), slice thickness, and convolution kernel are relevant sources of CT-radiomics variability.*

*• Voxel size resampling increased the mean percentage of robust CT-radiomics features from 59.50 to 89.25% when comparing CT scans acquired with different pixel sizes and from 71.62 to 82.58% when the scans were acquired with different slice spacings.*

*• ComBat batch effect correction reduced the CT-radiomics variability secondary to the slice thickness and convolution kernel, improving the capacity of CT-radiomics to differentiate tissues (in the phantom application) and the primary tumor type from liver metastases (in the clinical application).*

**Electronic supplementary material:**

The online version of this article (10.1007/s00330-020-07174-0) contains supplementary material, which is available to authorized users.

## Introduction

Radiomics is revolutionizing medical image assessment and interpretation, moving from a subjective evaluation to a quantifiable -omics image assessment method [1, 2]. Multiple studies have shown that radiomics provides meaningful information about cancer and correlates with histological and molecular tumor phenotypes, creating opportunities to develop novel predictive and prognostic biomarkers for cancer [3, 4]. The maximum benefit for cancer patients has been shown when tailoring treatments to specific cancer characteristics [5]. Thus, radiomics can play a key role in improving personalized medicine. However, radiomics features are influenced by the image-acquisition technique and the reconstruction parameters [6–9]. Studies performed at a single institution usually do not account for this source of variability, and then, the results entail low scalability of the signatures for multi-centric applications.

To achieve meaningful generalizable radiomics-based tools, large-scale studies are necessary [10]. These require multicenter data collection, which implies scans acquired with different protocols, particularly when including retrospective data. Different strategies have been followed to minimize the effects of radiomics variability. Aerts et al considered radiomics variability as a feature selection tool by using test–retest analysis, eliminating radiomics features with high variability based on their cohort results [5, 11]. Sun et al introduced the image-acquisition parameters as a confounding variable into the model [3], and Choe et al explored convolutional neural networks-based kernel conversion for reducing radiomics variability [12].

There is an unmet need to establish robust pre- or post-image-acquisition methods for radiomics data harmonization. In this study, we explore the main image-acquisition factors that generate radiomics variability. These variability-causing factors are called “batch effects” [13]. Different batch effect correction techniques have been developed, allowing for genomics and proteomics data harmonization [14]. These batch effect correction techniques aim to remove the variance of the signal caused by the variability between batches to improve the biological signal. Alter et al defined the singular value decomposition (SVD)-based batch effect removal, where the principal components associated with the batch variability are filtered from the data and the matrix is reconstructed without these factors [15]. Johnson et al implemented the ComBat algorithm for batch correction based on an empirical Bayes approach to standardize the means and variances across batches to reduce the batch effect error [16, 17]. However, there is little evidence of the application of these methods towards reducing radiomics variability [18].

In this study, we aim to describe the image-acquisition-based sources of CT-radiomics variability. We also explore the role of image resampling and batch effect as post-image-acquisition correction methods for reducing radiomics variability, thereby improving the classification accuracy of radiomics in phantom and clinical applications.

## Materials and methods

**Fig. 1:**
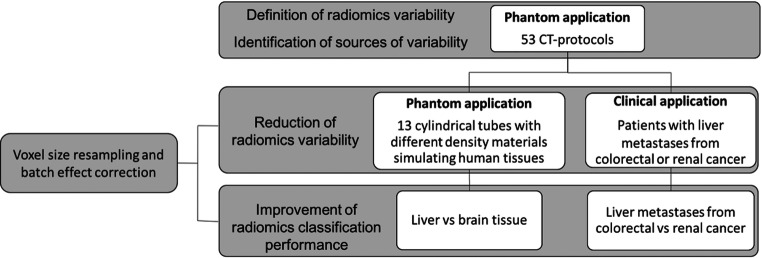
Methodology flowchart

Multiple images of a phantom were acquired with different CT-acquisition protocols to explore the radiomics variability and identify the sources of variability according to the CT-acquisition parameters. Then, image post-processing and batch correction methods were implemented to reduce the radiomics variability in phantom and clinical applications. Finally, we explored the improvement of radiomics classification performance by reducing the radiomics variability (Fig. 1).

The retrospective clinical study was approved by the institutional review board. Informed consent for the computational analysis of the CT images was waived.

### Phantom image acquisition

The Gammex Model 467 Tissue Characterization Phantom (Gammex RMI) was used to describe intra-scanner variability for different acquisition parameters. This phantom includes a matrix with 13 rods of 33 cm diameter with different density materials simulating human tissues (Supplementary Material 1).

Phantom CT scans were acquired in a 16-channel Philips CT scanner by fixing all the acquisition parameters except the one tested. The tested acquisition parameters included voltage, current, slice thickness, and voxel size (accounting for the slice spacing and the pixel size) with a total of 25 different acquisition protocols (Supplementary Material 2). The minimum and maximum values of the acquisition parameters (voltage, slice thickness, slice spacing, and pixel size) were reconstructed with all the available Philips-specific reconstruction kernels (A, B, C, D, and E) to study the kernel variability with different acquisition parameter sets. The rest was reconstructed with kernel “A,” leading to 53 different protocols (Supplementary Material 3).

### Clinical image acquisition

The clinical study included 43 patients (mean [range] age 66.41 [41–77] years; 46.51% [20/43] female, 53.49% [23/43] male) with 97 liver metastases (mean [range] lesions per patient 2.26 [1–7]) from colorectal adenocarcinoma (53.61% [52/97]) and clear cell renal carcinoma (46.39% [45/97]) [19].

Contrast-enhanced CT scans were collected retrospectively and acquired at Vall d’Hebron University Hospital and Bellvitge University Hospital between November 2013 and September 2019 with two specific acquisition protocols from General Electric and Siemens CT scanners. Additionally, all CT scans from the open-access database The Cancer Genome Atlas Kidney Renal Clear Cell Carcinoma (TCGA-KIRC) [20] were acquired with a General Electric scanner. Only patients with liver metastases from this open-access database were included in the analysis. Detailed information of CT-scan acquisition and reconstruction protocols for each hospital and the TCGA-KIRC database are defined in Supplementary Material 4.

### Image processing and radiomics features extraction

**Fig. 2:**
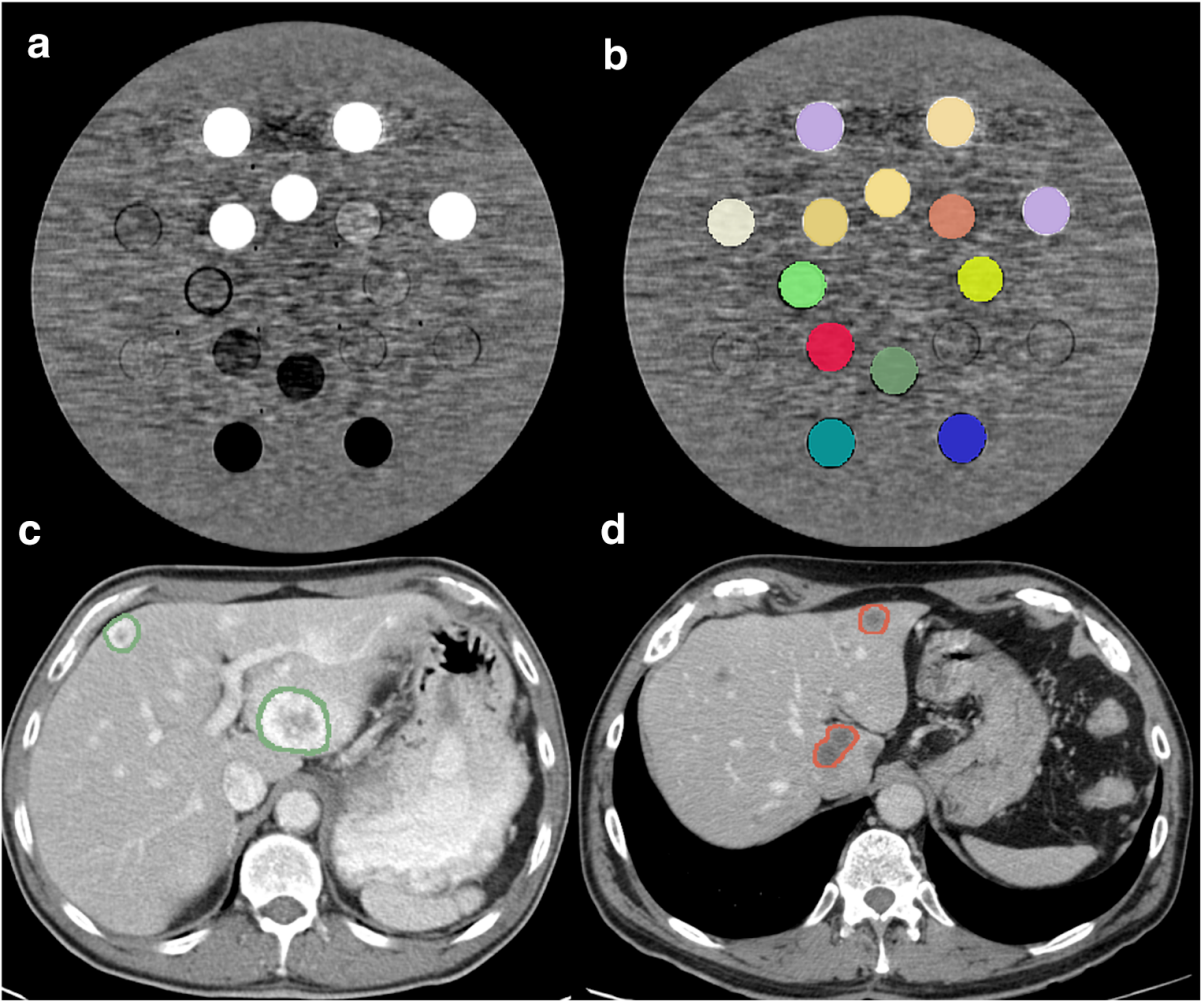
Axial CT of the Gammex 467 Tissue Characterization Phantom showing the thirteen tissue and water materials (**a**) with the segmented volumes of interest (VOI) for the different rod materials (**b**). Axial enhanced CT of the abdomen showing the target liver metastases (green and red masks) of a patient with clear cell renal carcinoma (**c**) and a patient with colorectal adenocarcinoma (**d**)

In the phantom application, the 13 rods were delineated (including the entire rod) with a semi-automatic contouring function from 3DSlicer v4.8.1 [21], obtaining one volume of interest (VOI) per rod. The same VOI per rod was used to extract the radiomics features from the phantom in all the studied CT-acquisition protocols (Fig. 2a, b). Image registration was not needed, given that the scans were acquired with the same starting and ending position, and the phantom’s position did not change between scans.

In the clinical application, all well-defined liver metastases were included in the analyses (Fig. 2c, d). Small metastases (i.e., largest diameter < 1 cm) or with artifacts were excluded. Lesions were delineated with the 3DSlicer v4.8.1 semi-automatic contouring function [21] supervised by a radiologist physician with 10-year experience in oncological imaging.

In the phantom application, radiomics data from images without voxel resampling were extracted to study the impact of image resampling on radiomics data. For batch correction analysis, the images and masks were resampled to isometric voxels of 1 × 1 × 1 mm^3^ using spline interpolation and nearest-neighbor interpolation, respectively. Image values were discretized to a bin size of 50 HU; afterwards, the CT-radiomics features from the VOIs were extracted. The radiomics features, including first-order and texture analyses, were derived using an in-house program based on the Pyradiomics package for Python [22]. For texture feature extraction, five gray-level matrices (gray-level co-occurrence matrix [GLCM], gray-level dependence matrix [GLDM], gray-level run length matrix [GLRLM], gray-level size zone matrix [GLSZM], and neighboring gray-tone different matrix [NGTDM]) were calculated in three dimensions. Ninety-three radiomics variables were obtained for each VOI, including variables from the first-order histogram and the five gray-level matrices (Supplementary Material 5).

### Sources of variability identification

Relevant sources of variability were identified by the intraclass correlation coefficient (ICC) of all the radiomics features between different acquisition parameters accounting for pixel size, reconstruction slice spacing (interpolated from the raw CT-image data without voxel resampling), acquisition slice thickness, convolution kernel, current, and voltage. The CT-acquisition variables that presented less than 80% of robust radiomics features (i.e., less than 80% of the features with ICC > 0.8 [23]) were defined as relevant sources of variability (batches) for further correction.

### Techniques for radiomics variability correction

#### Image resampling

To correct variability from parameters related to voxel size, radiomics data were extracted from images resampled to isometric voxels of 1 × 1 × 1 mm^3^. Acquisition voxel size variability was analyzed separately by pixel size and slice spacing. Radiomics data of all the phantom materials with different acquisition pixel sizes (0.35 × 0.35, 0.78 × 0.78, and 1 × 1 mm^2^) and slice spacings (1, 1.25, 2, 2.5, and 5 mm) were included while the rest of parameters remained fixed.

To assess the effect of resampling data on variability correction, the ICCs between groups of different acquisition pixel sizes and slice spacings were computed before and after resampling. Principal component analysis (PCA) was implemented to qualitatively show the reduction of variability on radiomics data variance caused by resampling the acquisition voxel size.

#### Batch effect removal

To correct variability sources related to image acquisition and reconstruction, two methods of batch correction were applied: singular value decomposition-based (SVD-based) correction [15] and ComBat correction [16]. ComBat correction was applied using the SVA package from R version 3.6.1. [17]. For SVD-based correction, principal components (PC) with higher correlation with batches (i.e., convolution kernel and slice thickness defined as per the ICC analysis) were removed from the PCA space, and the matrix was reconstructed back to the feature space (Supplementary Material 6). ComBat correction with parametric adjustments was applied three times considering the sources of variability as batches (i.e., convolution kernel and slice thickness and the slice thickness–convolution kernel combination).

To evaluate data correction, the ICCs between groups of acquisition parameters were assessed before and after the application of two different batch corrections. Principal component analysis (PCA) was implemented to qualitatively show the reduction of variability on radiomics data variance caused by batch correction techniques.

Two-proportion *z* test was applied to compare the percentage of robust features before and after correction. The *p* value threshold for significance was established at 0.05. Adjustment for multiple testing was performed by controlling the false discovery rate at 0.05 according to the Benjamini and Hochberg method.

### Validation analysis

Unsupervised *K*-means clustering purity was used to evaluate the improvement in data classification after batch variability correction with SVD and ComBat. To measure clustering purity, each cluster is assigned to the most frequent class in the cluster. Then, the accuracy is measured by counting the number of correctly classified data in the assigned class. The performance of the unsupervised clustering before and after the implementation of batch correction was analyzed in a phantom and a clinical application. The clustering performance was also analyzed in non-resampled data.

#### Phantom application

Two similar phantom materials (liver and brain) were included. Batch effect correction by ComBat was applied three times considering different sources of variability as batches: convolution kernel (five batches: A, B, C, D, E), slice thickness (three batches: 2, 3, 5 mm), and the combination of convolution kernel with slice thickness (15 batches; all possible combinations of convolutional kernel and slice thickness).

#### Clinical application

The clinical application aimed to analyze the performance of clustering different primary tumor types (colorectal versus renal) based on liver metastasis radiomics data. Batch effect correction by ComBat was applied three times considering different sources of variance as batches: manufacturer-dependent convolution kernel (two batches: General Electric and Siemens), slice thickness (four batches: 1.25, 2, 2.5, 5 mm), and the combination of convolution kernel with slice thickness (eight batches; all possible combinations of manufacturers and slice thickness).

The *K*-means clustering was computed 1000 times, and the highest purity of the clustering appearing on more than 20% of the iterations was chosen for the comparison between the initial data and the data after different batch correction techniques (SVD-based, ComBat) [24].

## Results

### Population (phantom and clinical applications)

In the phantom application, a total of 53 different CT scans of the 13 phantom materials were acquired in a Big Bore 16 CT scanner (Philips) with different acquisition parameters and reconstruction kernels.

The clinical population included 97 liver metastases from 43 patients. CT scans were retrospectively collected from Vall d’Hebron University Hospital (26/43) and Bellvitge University Hospital (12/43). In addition, five cases from the open-access database TCGA-KIRC were also included. CT images were acquired in CT scanners from two manufacturers: 60.46% (26/43) from Sensation 64 CT scanner (Siemens) and 39.53% (17/43) from Light Speed Pro 16 CT scanner (General Electric) (Table 1).

**Table 1:** Population description by tumor type (clinical application)

	Colorectal adenocarcinoma	Clear cell renal cancer	Total
*N* patients	24/43 (55.81%)	19/43 (44.19%)	43/43 (100%)
*N* patients Vall d’Hebron University Hospital	18/26 (69.23%)	8/26 (30.77%)	26/43 (60.47%)
*N* patients Bellvitge University Hospital	6/12 (50.00%)	6/12 (50.00%)	12/43 (27.90%)
*N* patients TCGA-KIRC	0/5 (0%)	5/5 (100%)	5/43 (11.63%)
*N* lesions	52/97 (53.61%)	45/97 (46.39%)	97/97 (100%)
*N* lesions Siemens	28/51 (54.90%)	23/51 (45.10%)	51/97 (52.58%)
*N* lesions GE	24/46 (52.17 %)	22/46 (47.83%)	46/97 (47.42%)

*TCGA-KIRC* The Cancer Genome Atlas Kidney Renal Clear Cell Carcinoma, *GE* General Electric

### Defined sources of variability

**Fig. 3:**
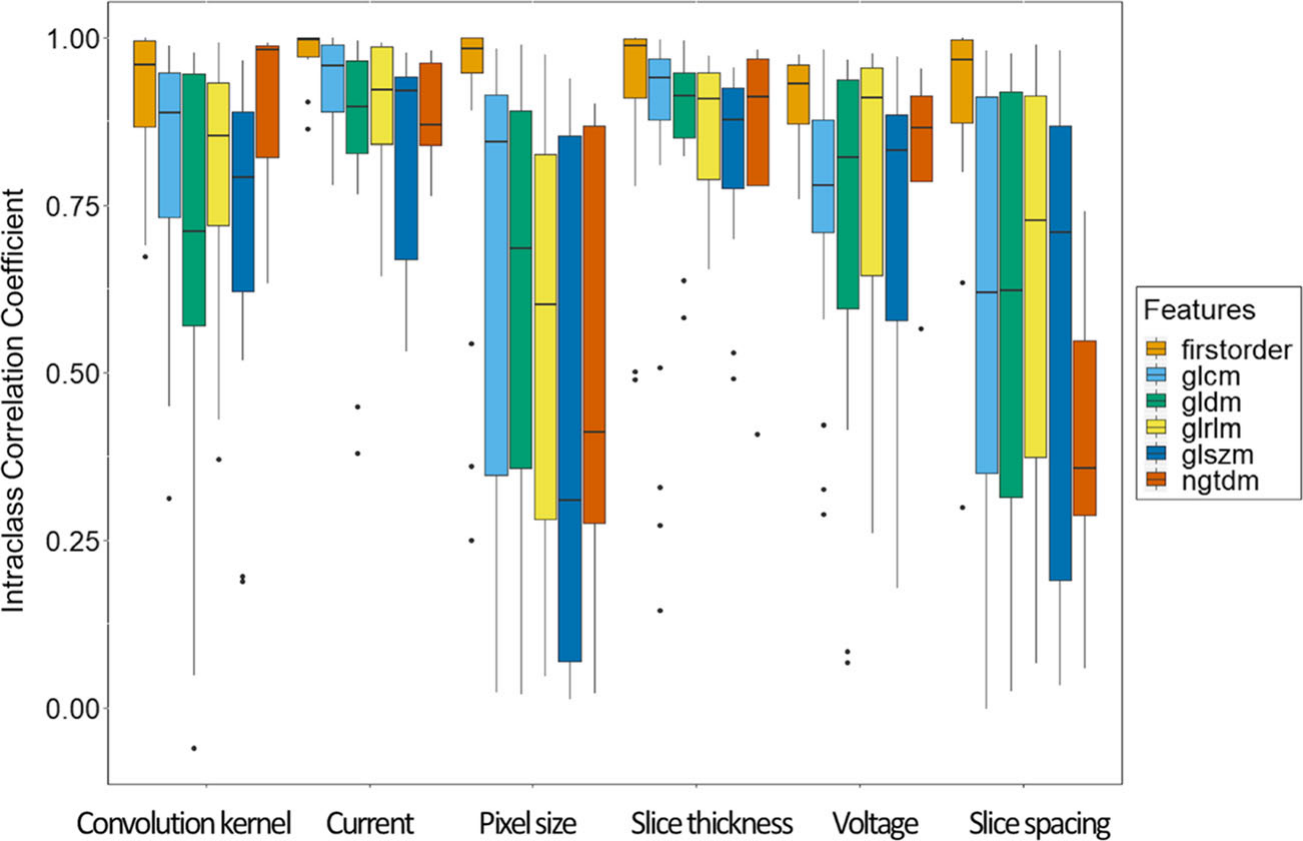
Intraclass correlation coefficients (ICCs) of the radiomics features of first order and texture matrices (gray-level co-occurrence matrix [GLCM], gray-level dependence matrix [GLDM], gray-level run length matrix [GLRLM], gray-level size zone matrix [GLSZM], neighboring gray-tone different matrix [NGTDM]) between extreme CT-acquisition parameters in the phantom application

For the phantom data, including all materials, ICCs between the different batches were assessed. Pixel size, slice spacing, slice thickness, convolution kernel, and voltage presented a low percentage of robust radiomics features (i.e., less than 80% of the radiomics features with ICC > 0.8) in at least one of the combinations from the ranging CT-acquisition parameters (Fig. 3).

Voxel size was defined as a relevant source of variability with a percentage of robust features ranging from 48.40 to 78.49% for pixel size and from 43.01 to 86.02% for slice spacing.

According to the slice thickness, the percentage of robust features ranged from 75.25% (when 2 mm and 5 mm were compared) to 88.17% (when 2 mm and 3 mm were compared). The percentage of robust radiomics features according to the convolution kernel ranged between 55.92% (when A and D were compared) and 97.85% (when A and B were compared).

The acquisition voltage of 90 kV showed the highest variability on radiomics data (65.59% of reproducible features). The standard voltage in clinical protocol range (i.e., 120–140 kV) showed higher radiomics robustness (81.72% of reproducible features). The percentages of robust features are described in Supplementary Material 7.

Therefore, voxel size, slice thickness, and convolution kernel were defined as the sources of variability with the highest impact on radiomics data reproducibility; voxel size was corrected by resampling, whereas slice thickness and convolution kernel were considered for batch correction.

### Evaluation of processing effects on radiomics data correction

#### Image resampling

**Fig. 4:**
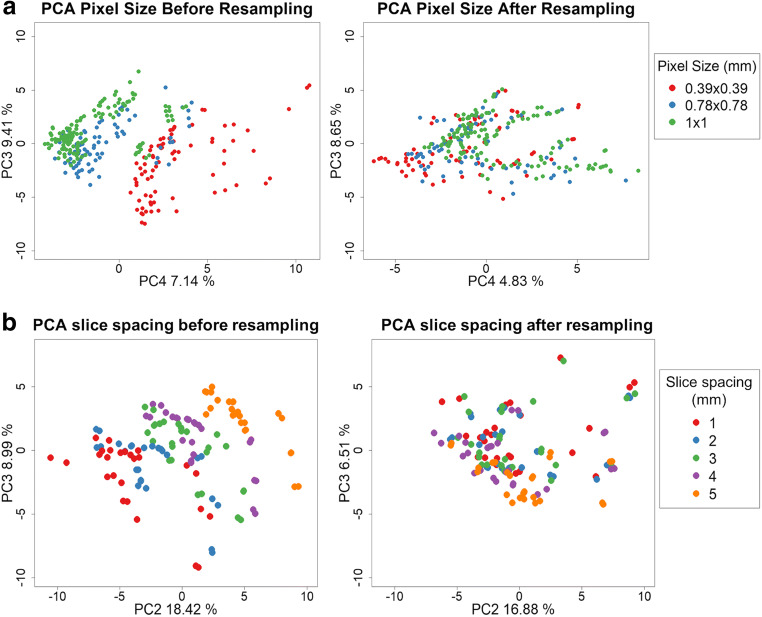
Principal component analysis (PCA) before and after resampling to 1 × 1 × 1 mm^3^ voxels of CT images acquired with different pixel sizes (**a**) and slice spacings (**b**). PC4 (explaining 7.14% of the radiomics data variance) is associated with the different acquisition pixel sizes before resampling. PC2 (18.42%) is associated with the distribution of the different acquisition pixel heights. After resampling, the acquisition voxel size (accounting for pixel size and slice spacing) is not associated with the variance explained by the PCA

 Voxel size resampling increased the mean percentage of robust features from 59.50 to 89.25% for pixel size and from 71.62 to 82.58% for slice spacing (Fig. 4). The percentage of robust radiomics features (ICC > 0.8) before and after resampling data to isometric voxels of 1 × 1 × 1 mm^3^ are defined in Table 2.

**Table 2:** Effect of voxel resampling to 1 × 1 × 1 mm^3^ on the percentage of robust features when comparing radiomics data from phantom CT scans with different voxel sizes. The percentage of robust radiomics features was compared before and after correction using two-proportion *z* test (*p* value < 0.05 in italics)

% reproducible features (ICC > 0.8) comparing CT scans of the phantom with different acquisition parameters			
Pixel size (mm^2^)	Non-resampled data	Pixel size resampled data 1 × 1 mm^2^	*p* value*
0.39 × 0.39–0.78 × 0.78	51.61 (48/93)	87.10 (81/93)	*< 0.01*
0.78 × 0.78–1 × 1	78.49 (73/93)	94.62 (88/93)	*< 0.01*
0.39 × 0.39–1 × 1	48.40 (45/93)	86.02 (80/93)	*< 0.01*
Slice spacing (mm)	Non-resampled data	Slice spacing resampled data 1 mm	*p* value
1–1.25	86.02 (80/93)	88.17 (82/93)	0.83
1.25–2	79.60 (74/93)	98.92 (92/93)	*< 0.01*
2–2.5	83.87 (78/93)	87.09 (81/93)	0.83
2.5–5	65.59 (61/93)	82.80 (77/93)	*0.03*
1–5	43.01 (40/93)	55.91 (52/93)	0.18

*ICC* intraclass correlation coefficient

*Adjustment for multiple testing was performed in each variability factor by controlling the false discovery rate according to the Benjamini and Hochberg method

#### Batch effect removal

Batch effect removal was implemented in all phantom materials based on the previously defined sources of variability (slice thickness and convolution kernel). For SVD-based batch correction, from the principal component analysis (PCA), PC2 significantly associated with convolution kernel (*p* < 0.001), and PC3 and PC4 significantly associated with slice thickness (*p* < 0.001); these principal components were removed at the transformed space, and the data matrix was back reconstructed to the feature space. The SVD-based correction technique increased the mean number of robust features (Table 3). Importantly, when analyzing images with different slice thicknesses, the mean number of robust radiomics features increased: from 82.79% without correction to 92.83% with SVD-based batch correction. When analyzing images restructured with different convolutional kernels, the mean percentage of robust radiomics features increased: from 78.45% without correction to 85.25% with SVD-based correction (Table 3, Supplementary Material 8).

**Table 3:** Percentage of robust radiomics features (ICC > 0.8) without batch correction and after batch correction with singular value decomposition (SVD) and ComBat methods. The percentage of robust radiomics features was compared before and after correction using two-proportion *z* test (*p* value < 0.05 in italics)

	% Reproducible features comparing CT scans of the phantom with different acquisition parameters						
	Initial data	Batch effect correction methods					
		SVD Thickness–kernel	SVD Kernel	SVD Thickness	ComBat Thickness–kernel	ComBat Kernel	ComBat Thickness
Slice thickness (mm)							
2–3	88.17 *p* value**	97.85 0.06	96.77 0.16	98.92* *0.01*	97.85 *0.026*	94.62 0.28	97.85 *0.02*
3–5	84.95 *p* value	93.55 0.10	88.17 1	96.77* *0.01*	95.70 *0.026*	93.55 0.28	96.77* *0.02*
2–5	75.27 *p* value	87.10 0.09	75.27 1	90.32 *0.01*	92.47* *0.008*	82.80 0.28	89.25 *0.02*
Convolution kernel							
A–B`	97.85 *p* value	98.92* 1	97.85 1	98.92* 1	97.85 1	97.85 1	96.77 1
B–C	93.55 *p* value	95.70 0.87	94.62 1	95.70 1	98.92* 0.14	96.77 0.58	92.47 1
C–D	72.04 *p* value	81.72 0.38	78.49 0.62	78.49 1	86.02* *0.07*	86.02* *0.05*	73.12 1
D–E	79.57 *p* value	86.02 0.58	84.95 0. 62	82.80 1	90.32 0.11	91.40* *0.05*	78.49 1
A–E	83.87 *p* value	88.17 0.74	89.25 0.390	83.87 1	92.47 0.14	95.70* *0.04*	82.79 1
B–D	62.37 *p* value	75.27 0.21	73.11 0. 62	64.51 1	81.72 *0.01*	83.87* *< 0.01*	61.29 1
D–A	55.91 *p* value	70.97 0.21	65.59 0. 62	62.37 1	79.57 *0.01*	83.87* *< 0.01*	59.13 0.767

*ICC* intraclass correlation coefficient, *SVD* singular value decomposition

*The highest increase of robust radiomics features

**Adjustment for multiple testing was performed in each variability factor and correction method by controlling the false discovery rate according to the Benjamini and Hochberg method

The ComBat correction technique increased the mean percentage of robust features to 95.34% for slice thickness and to 89.55% for convolution kernel when considering as batches the convolution kernel–slice thickness combination (Table 3, Supplementary Material 8).

### Improvement in *K*-means clustering performance

To test the classification performance based on radiomics features and the potential improvement by reducing radiomics variability by batch correction, a classification of similar density tissues was performed.

#### Phantom application

**Fig. 5:**
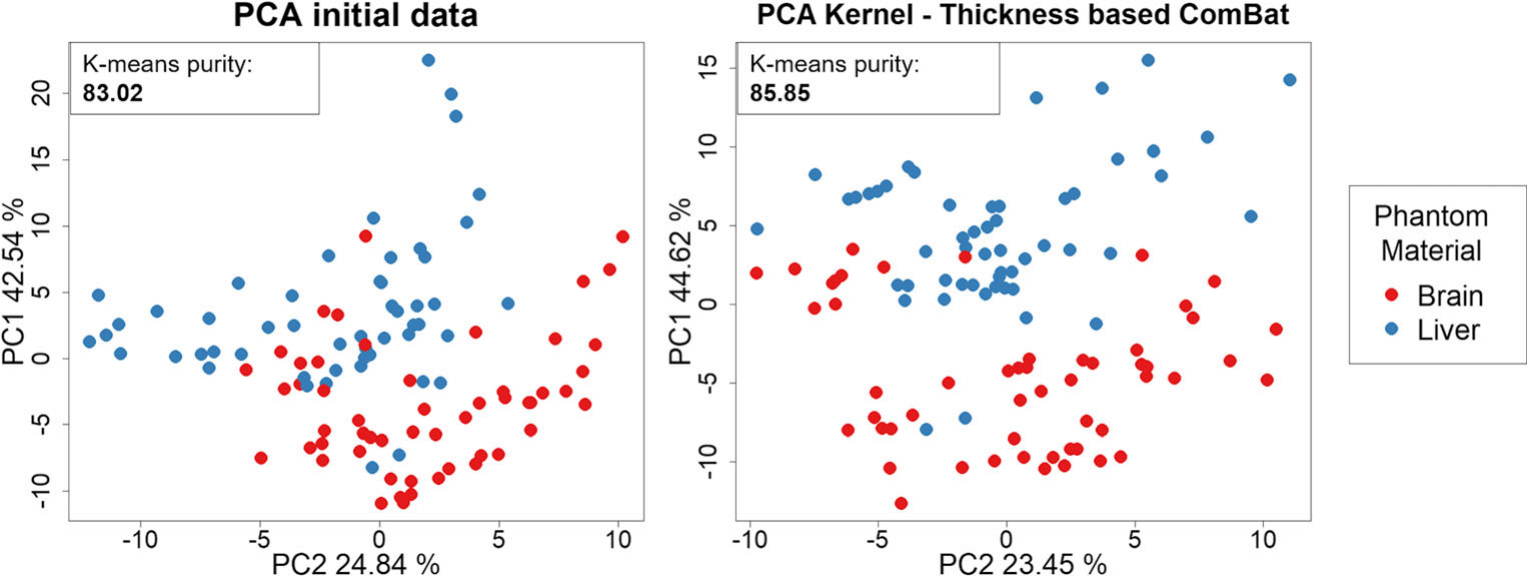
Principal component analysis (PCA) of the brain and liver material radiomics distribution before and after convolution kernel–slice thickness ComBat correction. The distance between the radiomics data of the brain and liver materials from CT scans with different acquisitions protocols increases after applying batch correction (i.e., the radiomics distribution better reflects differences between materials and not due to the CT-acquisition parameters)

The phantom application included liver and brain phantom tissues. SVD-based correction was applied, removing the PC2 (24.84%) and PC3 (13.53%), which associated significantly with slice thickness (*p* < 0.001), and PC5 (3.21%) that associated significantly with convolution kernel (*p* < 0.001). ComBat was applied three times for batch correction, defined as convolution kernel, slice thickness, and convolution kernel–slice thickness combination. The *K*-means purity for tissue classification after SVD and ComBat batch correction is described in Table 4. Importantly, ComBat correction considering convolution kernel–slice thickness combination as batch effects showed the highest clustering purity (85.85%) (Fig. 5).

**Table 4:** *K*-means purity for phantom tissue (brain vs liver) and tumor type (colorectal carcinoma vs clear cell renal carcinoma) classification before and after batch correction

	*K*-means purity						
	Initial data	SVD Thickness–kernel	SVD Kernel	SVD Thickness	Combat Thickness–kernel	Combat Kernel	Combat Thickness
Phantom	83.02	78.30	78.30	85.85*	85.85*	84.90	82.07
Tumor type	65.98	62.89	62.89	62.89	67.01	73.20*	67.01

*SVD* singular value decomposition

*The highest improvement of *K*-means purity classification

In the phantom study, the clustering performance from resampled images did not show improvement from non-resampled image data clustering (Supplementary Material 9).

#### Clinical application

In the clinical application of tumor type classification based on liver metastasis, the SVD was applied removing the PC1 (34.21%) that associated significantly with slice thickness and convolution kernel (*p* < 0.001). Batch correction by ComBat was applied three times, considering as batches the convolution kernel, slice thickness, and convolution kernel–slice thickness combination.

**Fig. 6:**
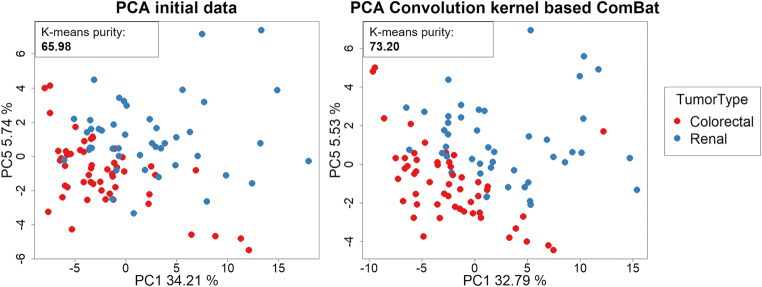
Principal component analysis (PCA) of the liver metastasis radiomics distribution from CT scans of patients with colorectal adenocarcinoma and clear cell renal carcinoma. PCA before and after convolution kernel ComBat correction. The distribution of the groups of patients with different tumor types differs more after batch correction. The first component (PC1 [%]) of data variance can differentiate better between groups (colorectal versus renal) after correction

The *K*-means improvement for tissue classification after SVD and ComBat batches correction are described in Table 4. Importantly, ComBat correction for the convolution kernel showed the best performance for primary tumor type classification based on radiomics data from liver metastasis (purity = 73.20%) (Fig. 6).

In the clinical application, the clustering performance from resampled images did not show improvement from non-resampled image data clustering (Supplementary Material 9).

## Discussion

The capacity to extract a large amount of valuable quantitative data from medical images, such as CT, is revolutionizing the way medical scans can be evaluated. However, the development of reliable imaging biomarkers requires robust CT-based radiomics data. In this study, we defined the main sources of CT-radiomics variability based on a comprehensive phantom study with multiple CT-acquisition protocols. We also evaluated the influence of image resampling and the effect of radiomics data normalization by means of batch effect correction to reduce the variability and improve the tissue-classification capacity of radiomics in a phantom and clinical application.

We have shown that voxel size, convolution kernel, and slice thickness are relevant sources of variability. The voxel size has the highest impact on radiomics variability, particularly on texture features. Convolution kernel also affects first-order features, which are overall the most robust features regardless of the CT-acquisition protocol. Similarly to Berenguer et al [8], we also found that the radiomics features presented more variability when evaluated in CT scans acquired with low voltage values (90 kV). Importantly, the radiomics features were more robust when the voltage was within the range applied in standard clinical practice (i.e., 120–140 kV).

The pixel size varies in each scan and for each patient due to the changing field of view, limiting the possibility to pre-define this parameter. This study shows that image processing techniques regarding voxel resampling reduce the variability caused by the acquisition voxel size. A possible explanation for this variability decrease could be the resolution homogenization and the smoothness in gray-level transitions in the resampling direction. Therefore, in the *z*-direction, despite that we cannot restore the missing information in a large voxel size (e.g., 5 mm), when resampling, the texture analysis considers the changes in gray levels inside the original voxel size and avoids abrupt gray-level changes to make them more comparable to the *x*- and *y*-directions.

In order to correct the variability caused by the reconstruction kernel and acquisition slice thickness, SVD-based and ComBat batch correction techniques were applied to radiomics data considering both image parameters as batches. In line with Orlhac et al [18], we demonstrate that after applying ComBat, the distribution of the data (using PCA) was modified to differentiate the phantom materials based on radiomics features. In our study, we also show that the reproducibility improves by means of ICCs. However, we aimed to study not only the variability correction by ComBat but also how the tissue-classification performance of radiomics improves after this variability correction. We have shown that ComBat correction for kernel and for kernel–slice thickness combinations in both the phantom and clinical applications outperforms the classification accuracy of radiomics data. The SVD-based correction improved the reproducibility of the radiomics features, although this could have suffered from overcorrection, leading to a loss of biological meaning and decreasing the tissue-clustering accuracy of radiomics.

Deep learning techniques have been developed to reduce radiomics variability by reconstructing images to the same convolution kernels [12]. However, the need for large data sets and the wide variety of intra- and inter-manufacturer reconstruction kernels limits the application of these techniques. ComBat correction can be applied in smaller datasets due to the non-parametric adjusting methods used to correct data variance associated to a particular factor.

The results of our study are promising, but we acknowledge some limitations. First, we implemented a variability correction method in both phantom and clinical applications. The phantom was used to assess the intra-scanner variability from one CT vendor while the clinical application analyzed the inter-scanner variability for ComBat and SVD-based correction. Further studies with intra- and inter-manufacturer CT scans could be performed to extend the application of batch correction methods. Moreover, there are several reconstruction kernels along manufacturers that could be considered comparable, as proposed by Mackin et al [25]. This would reduce the inter-manufacturer variability and would facilitate the definition of batches based on inter-manufacturer similar kernels for large-scale multicenter studies. Second, the radiomics variability correction was clinically tested as a method to improve the tumor type classification. Although this highlights the impact of post-acquisition CT-radiomics normalization by means of batch correction, further applications need to be tested and validated in larger populations. Finally, the described normalization methods have been tested in CT images; it is of interest to test these in multi-image modalities including MRI.

In conclusion, the main sources of CT-radiomics variability are slice thickness and reconstruction kernels. The application of image post-processing and the ComBat correction method minimizes radiomics data variability regardless of the differences in the CT-image-acquisition protocols. These methods are easy to apply to expand the potential of radiomics implementation in new retrospective and prospective multicenter large-scale studies where the variability of the acquisition protocols and scanners is the major limitation.

## Electronic supplementary material

ESM 1 (DOCX 292 kb)

## Abbreviations

GLCM: Gray-level co-occurrence matrix
GLDM: Gray-level dependence matrix
GLRLM: Gray-level run length matrix
GLSZM: Gray-level size zone matrix
ICC: Intraclass correlation coefficient
NGTDM: Neighboring gray-tone different matrix
PCA: Principal component analysis
SVD: Singular value decomposition
TCGA-KIRC: The Cancer Genome Atlas Kidney Renal Clear Cell Carcinoma
VOI: Volume of interest
