# Supplementary material for: Minimizing acquisition-related radiomics variability by image resampling and batch effect correction to allow for large-scale data analysis
Source: Eur Radiol. 2020 Sep 9;31(3):1460–70. doi: 10.1007/s00330-020-07174-0 (PMC7880962; doi:10.1007/s00330-020-07174-0)
Supplement: Supplementary file 1 — (DOCX 292 kb) [file 330_2020_7174_MOESM1_ESM.docx]

**Supplementary Material 1. Physical Densities and the Electron Densities of tissue and water materials.**

| **Rod Material** | **Electro Density**  **Relative to Water** | **Physical Density (gm/cm^3^)** |
| --- | --- | --- |
| Lung (LN-300) | 0.29 | 0.30 |
| Lung (LN-450) | 0.40 | 0.45 |
| Adipose (AP6) | 0.90 | 0.92 |
| Breast | 0.96 | 0.99 |
| CT Solid Water | 0.99 | 1.02 |
| Brain | 1.05 | 1.05 |
| Liver | 1.07 | 1.08 |
| Inner Bone | 1.09 | 1.12 |
| Bone (B200) | 1.11 | 1.15 |
| Bone (CB2 – 30% Mineral) | 1.28 | 1.34 |
| Bone (CB2 – 50% Mineral) | 1.47 | 1.56 |
| Cortical Bone (SB3) | 1.69 | 1.82 |
| True Water | 1.00 | 1.00 |

**Supplementary Material 2. Image acquisition and reconstruction parameters in the phantom study.**

| **Voltage (kV)** | 90 | | | 120 | | 140 |
| --- | --- | --- | --- | --- | --- | --- |
| **Current (mA)** | 100 | | 200 | 300 | 400 | 500 |
| **Slice Thickness (mm)** | 2 | | | 3 | | 5 |
| **Slice spacing (mm)** | 1 | 1.25 | | 2 | 2.5 | 5 |
| **Pixel Size (mm)** | 0.3 | 0.58 | | 0.78 | 0.97 | 1 |
| **Reconstruction Kernel** | A | B | | C | D | E |

**Supplementary Material 3. CT acquisition protocols in the phantom study.**

Different acquisition parameters were considered for the phantom reproducibility study (see table, Supplemental Material 2 which illustrates image acquisition and reconstruction parameters for Phantom analysis). A total of 25 different scans were acquired combining the different parameters from Supplemental Material 2. First, all the combinations between voltage and current were acquired to analyze the relation between these acquisition variables with a total of 15 acquisition. The rest of the images were acquired by fixing all the parameters but the tested one. The parameters were set to a standard acquisition with a voltage of 120kV, X ray tube current of 300 mA and a slice thickness of 2 mm. The Field of View (FOV) was set to 512 mm with a matrix of 512x512 leading to a pixel size of 1x1 mm^2^. The spacing between slices was set to 1 mm to obtain isometric voxels of 1mm^3^. All the acquisitions were reconstructed with the Philips-specific reconstruction kernel ‘A’. Therefore, three different acquisitions were obtained for slice thickness and five different acquisition were obtained for spacing between slices and pixel size. Considering that two acquisition parameter set were repeated, the total number of acquisitions were 25 and not 28 scans.

To study the variability between reconstruction kernels with a diversity of acquisition parameters, the acquisitions with the minimum and the maximum values of voltage (90-140 kV), slice thickness (2 – 5 mm), spacing between slices (1 – 5 mm) and pixel size (0.38 – 1 mm) were reconstructed with all the Philips-specific kernels (‘A’, ‘B’, ‘C’, ‘D’, ‘E’). In case of the pixel size, the median value (0.78 mm) was also reconstructed with the different kernels. Therefore, there are nine acquisitions with 4 extra different kernels (‘B’, ‘C’, ‘D’, ‘E’). Considering that two of these acquisitions are repeated, seven acquisitions with kernels ‘B’, ‘C’, ‘D’ and ‘E’ are added to the initial number of acquisition (n = 25), leading to a final number of 53 acquisitions.

**Supplementary Material 4. Clinical application CT-scans acquisition and reconstruction parameters from each hospital and The Cancer Genome Atlas Kidney Renal Clear Cell Carcinoma (TCGA-KIRC).**

| **Tumor type** | **Vall d’Hebron University Hospital** | **Bellvitge University Hospital** | **TCGA-KIRC** |
| --- | --- | --- | --- |
| **N** | **26** | **12** | **5** |
| **Siemens** |  |  |  |
| I31f | 8/26 | 0 | 0 |
| B30f | 14/25 | 4/12 | 0 |
| **GE** |  |  |  |
| STANDARD | 4/26 | 8/12 | 5/5 |
| **Slice Thickness** |  |  |  |
| **<5 mm** | 12/26 | 8/12 | 3/5 |
| **5 mm** | 14/26 | 4/12 | 2/5 |
| **Voltage**  Median [range] | 120 [110-120] | 120 [120-120] | 120 [120-120] |
| **Current**  Median [range] | 319 [104-580] | 238 [103-378] | 381 [235-440] |
| **Slice spacing**  Median [range] | 2.5 [1-5] | 2.5 [2.5-5] | 2.5 [2.5-5] |
| **Pixel spacing**  Median [range] | 0.857  [0.701-0.977] | 0.899  [0.750-0.977] | 0.789  [0.703-0.918] |

Abbreviations: Hospital 1(XX1), Hospital 2 (XX2), The Cancer Genome Atlas Kidney Renal Clear Cell Carcinoma (TCGA-KIRC).

**Supplementary Material 5. Radiomics features from first order and texture analysis.**

| **First-order** | | | |
| --- | --- | --- | --- |
| 10 Percentile | Mean Absolute Deviation | Root Mean Squared | Skewness |
| 90 Percentile | Median | Uniformity | Total Energy |
| Energy | Minimum | Kurtosis | Variance |
| Robust Mean Absolute Deviation | Range | Maximum |  |
| Interquartile Range | Entropy | Mean |  |
| **GLCM** | | | |
| Autocorrelation | Difference Average | Idn | Maximum Probability |
| Cluster Prominence | Difference Entropy | Imc1 | Sum Average |
| Cluster Shade | Difference Variance | Inverse Variance | Sum Entropy |
| Cluster Tendency | Id | Joint Average | Sum Squares |
| Contrast | Idm | Joint Energy |  |
| Correlation | Idmn | Joint Entropy |  |
| **GLDM** |  |  |  |
| Dependence Entropy | Gray Level Non-Uniformity | Large Dependence High Gray Level Emphasis | Small Dependence High Gray Level Emphasis |
| Dependence Non-Uniformity | Gray Level Variance | Large Dependence Low Gray Level Emphasis | Small Dependence Low Gray Level Emphasis |
| Dependence Non-Uniformity Normalized | High Gray Level Emphasis | Dependence Variance |  |
| Low Gray Level Emphasis | Large Dependence Emphasis | Small Dependence Emphasis |  |
| **GLRLM** |  |  |  |
| Short Run Emphasis | Long Run Emphasis | Gray Level Variance | Run Variance |
| Gray Level Non-Uniformity Normalized | Long Run High Gray Level Emphasis | Run Length Non-Uniformity Normalized | Long Run Low Gray Level Emphasis |
| High Gray Level Run Emphasis | Gray Level Non-Uniformity | Run Length Non-Uniformity | Run Entropy |
| Short Run High Gray Level Emphasis | Low Gray Level Run Emphasis | Run Percentage | Short Run Low Gray Level Emphasis |
| **GLSZM** |  |  |  |
| Large Area Low Gray Level Emphasis | Small Area High Gray Level Emphasis | Size Zone Non-Uniformity | Small Area Low Gray Level Emphasis |
| Gray Level Non-Uniformity Normalized | Large Area High Gray Level Emphasis | Gray Level Non-Uniformity | Size Zone Non-Uniformity Normalized |
| Gray Level Variance | Zone Entropy | Small Area Emphasis | Zone Percentage |
| High Gray Level Zone Emphasis | Low Gray Level Zone Emphasis | Large Area Emphasis | Zone Variance |
| **NGTDM** |  |  |  |
| Busyness | Contrast | Strength | Complexity |
| Coarseness |  |  |  |

*Abbreviations*: Gray Level Co-occurrence Matrix (GLCM), Gray Level Dependence Matrix (GLDM), Gray Level Run Length Matrix (GLRLM), Gray Level Size Zone Matrix (GLSZM), Neighboring Gray Tone Difference Matrix (NGTDM)

**Supplementary Material 6. ComBat and Singular Value Decomposition-based correction methodology**

The ComBat algorithm assumes that batch effects can be modeled out by standardizing means and variances across batches. It models the value Y_ijf_ from feature f from sample j in batch I, with the following equation:

$$Y_{ijf}^{*}= Y_{ijf}-\alpha_{f}-X\beta_{f}-\gamma_{if}+{\delta_{if}\varepsilon}_{ijf}$$

Where $\alpha_{f}$ is the overall feature value, X is a design matrix for sample conditions, and $\beta_{f}$ is the vector of regression coefficients corresponding to X. The error ε_ijf_ is expected to follow a normal distribution with mean 0 and variance σ_f_^2^. The $\gamma_{if}$ and $\delta_{if}$ represent the additive and multiplicative batch effects of batch $i$ for features $f$. The adjusted values are obtained as:

$$Y_{ijf}^{*}= \frac{Y_{ijf}-\hat{\alpha_{f}}-X\hat{\beta_{f}}-\hat{\gamma_{if}}}{\delta_{if}}+\hat{\alpha_{f}}+X\hat{\beta_{f}}$$

Where $\hat{\alpha_{f}}$,$\hat{\beta_{f}}$ and $\hat{\gamma_{if}}$ are the estimated parameters from the model.

However, the difference between ComBat and previous batch effect correction methods is the use of Empirical Bayes (EB) to estimate the parameters of the modeling. Then, the EB estimates are used to adjust the data for batch effects (1).

In this study, the application of ComBat has been performed using the ComBat function from SVA package (R version 3.6.1) (2)

Singular Value Decomposition (SVD) is used for batch effect removal by computing the eigenvectors of the data, filtering out those factors that are associated with the variability components and reconstructing the matrix of data.

In this study, the principal component analysis (PCA) has been computed for the radiomics data and the first five principal components have been correlated with the different sources of variability (Slice thickness and Convolution kernel). This correlation was assessed using the square of the Pearson correlation coefficient (R^2^) from linear models between principal components and batch effects. Principal components with significant correlation with batches were removed and the matrix was reconstructed to the radiomics feature space.

**Supplementary Material 7. Percentage of robust features (intraclass correlation coefficient [ICC] >0.8) for the combinations of ranging and extreme CT-acquisition parameters.**

| **% Reproducible Features (ICC>0.8)** | |
| --- | --- |
| **Pixel size (mm^2^)** | |
| 0.39x0.39 – 0.78x0.78 | 51.61% (48/93) |
| 0.78x0.78 – 1x1 | 78.49% (73/93) |
| 0.39x0.39 – 1x1 | 48.40% (45/93) |
| **Slice spacing (mm)** | |
| 1 – 1.25 | 86.02% (80/93) |
| 1.25 – 2 | 79.60% (74/93) |
| 2 – 2.5 | 83.87% (78/93) |
| 2.5 – 5 | 65.59% (61/93) |
| 1 – 5 | 43.01% (40/93) |
| **Slice Thickness (mm)** | |
| 2-3 | 88.17% (82/93) |
| 3-5 | 84.95% (79/93) |
| 2-5 | 75.27% (70/93) |
| **Convolution Kernel** | |
| A-B | 97.85% (91/93) |
| B-C | 93.55% (87/93) |
| C-D | 72.04% (67/93) |
| D-E | 79.57% (74/93) |
| A-E | 83.87% (78/93) |
| B-D | 62.37% (57/93) |
| A-D | 55.91% (52/93) |
| **X-RayTube (mA)** | |
| 100 – 200 | 86.02% (80/93) |
| 200 – 300 | 87.10% (81/93) |
| 300 – 400 | 88.17% (82/93) |
| 400 – 500 | 98.92% (92/93) |
| 200 – 500 | 86.02% (80/93) |
| **Voltage (kV)** | |
| 90-120 | 69.89% (65/93) |
| 120-140 | 81.72% (76/93) |
| 90-140 | 65.59% (61/93) |

**Supplementary Material 8. PCA before and after SVD and ComBat correction for Principal Components correlated with Slice Thicknes (2-3-5mm) and Convolutional Kernel (A and D)**


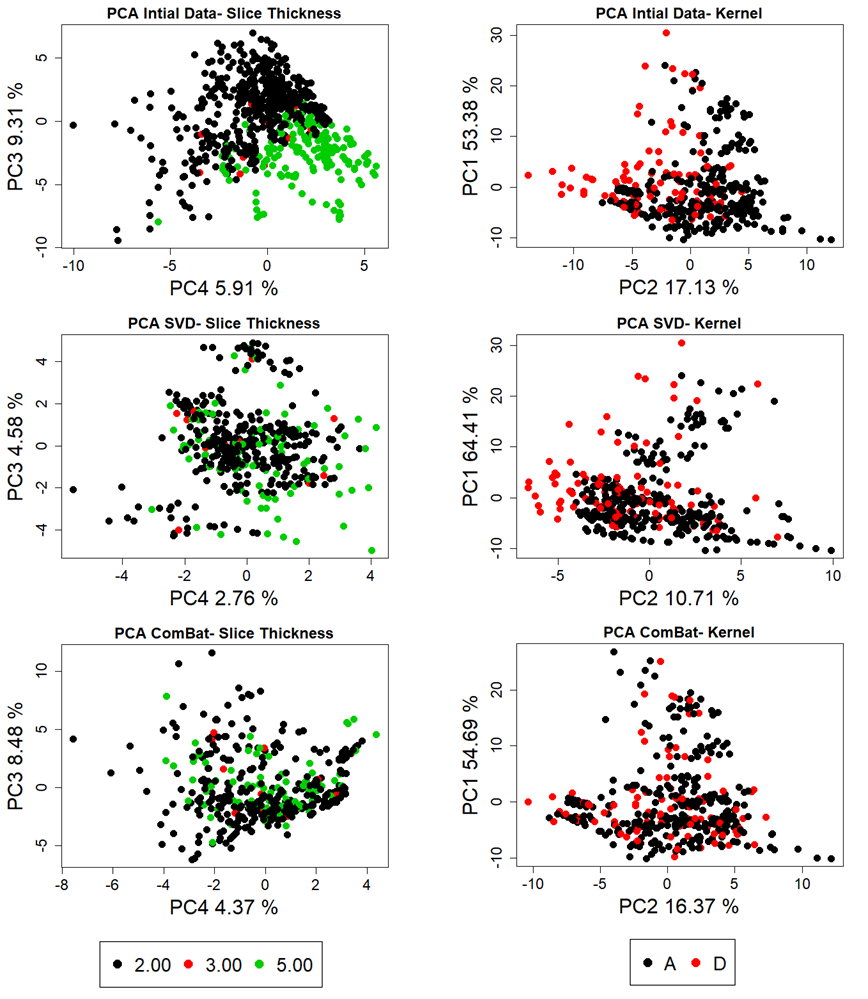


**Supplementary Material 9: Clustering performance for data with and without isometric voxel resampling in a Phantom and Clinical application.**

|  | **K-means purity** | |
| --- | --- | --- |
|  | **Non-resampled images** | **Resampled images** |
| **Phantom** | 83.96 | 83.02 |
| **Tumor Type** | 65.98 | 65.98 |

1. Johnson WE, Li C, Rabinovic A**.** Adjusting batch effects in microarray expression data using empirical Bayes methods. Biostatistics. 2007;8(1):118-27.

2. Leek JT, Johnson WE, Parker HS, et al. The sva package for removing batch effects and other unwanted variation in high-throughput experiments. Bioinformatics. 2012;28(6):882-3.
